# The Cognitive Symptom Checklist-Work in cancer patients is related with work functioning, fatigue and depressive symptoms: a validation study

**DOI:** 10.1007/s11764-015-0500-9

**Published:** 2015-11-30

**Authors:** H. F. Dorland, F. I. Abma, C. A. M. Roelen, A. Smink, M. Feuerstein, B. C. Amick, A. V. Ranchor, U. Bültmann

**Affiliations:** Department of Health Sciences, Community and Occupational Medicine, University Medical Center Groningen, University of Groningen, Antonius Deusinglaan 1, FA10, Room 616, 9713 AV Groningen, The Netherlands; Department of Health Psychology, University Medical Center Groningen, University of Groningen, Groningen, The Netherlands; ArboNed Occupational Health Services, Utrecht, The Netherlands; Department of Medical and Clinical Psychology and Preventive Medicine and Biometrics, The Uniformed Services University of the Health Sciences, Bethesda, MD USA; Department of Health Policy and Management, Robert Stempel College of Public Health and Social Work, Florida International University, Miami, FL USA; Institute for Work & Health, Toronto, ON Canada

**Keywords:** Cancer, Oncology, Cognitive symptom checklist, Work functioning, Internal consistency, Validity

## Abstract

**Purpose:**

The study objectives are to translate the 21-item Cognitive Symptom Checklist-Work (CSC-W21) to Dutch (CSC-W DV) and to validate the CSC-W DV in working cancer patients.

**Methods:**

The CSC-W21 was cross-culturally translated and adapted to a Dutch version. In this 19-item version, the dichotomous response option was changed to an ordinal five-point scale. A validation study of the CSC-W DV was conducted among cancer patients who had returned to work during or following cancer treatment. Internal consistency (Cronbach’s *α*), structural validity (exploratory factor analysis) and construct validity (hypothesis testing) were evaluated.

**Results:**

In a cohort of 364 cancer patients, 341 (94 %) completed the CSC-W DV (aged 50.6 ± 8.6 years, 60 % women). Exploratory factor analysis revealed two subscales ‘working memory’ and ‘executive function’. The internal consistency of the total scale and subscales was high (Cronbach’s *α* = 0.93–0.95). Hypothesis testing showed that self-reported cognitive limitations at work were related to work functioning (*P* < 0.001), fatigue (*P* = 0.001) and depressive symptoms (*P* < 0.001), but not to self-rated health (*P* = 0.14).

**Conclusions:**

The CSC-W DV showed high internal consistency and reasonable construct validity for measuring work-specific cognitive symptoms in cancer patients. The CSC-W DV was associated in expected ways with work functioning, fatigue and depressive symptoms.

**Implications for Cancer Survivors:**

It is important to enhance knowledge about cognitive symptoms at work in cancer patients, to guide and support cancer patients as good as possible when they are back at work and to improve their work functioning over time.

## Background

Cognitive symptoms are common among cancer patients and persist over time [1]. Cognitive symptoms can be attendant to the tumour or a result of treatment [1]. While 64 % return to work within 6 months after diagnosis [2], cancer patients can experience a reduced quality of life and function due to cognitive symptoms [1, 3]. Cognitive symptoms are associated with fatigue, depression and perceived health status [4–6]. Additionally, cognitive symptoms or function are negatively related to job performance, work ability, productivity and sustainable work [3, 7–12]. In the Netherlands, there is no native or cross-culturally adapted and validated self-report measure of cognitive functioning in the context of work for cancer patients available.

Cognitive deficits are usually identified by neuro-psychological assessment [13]. To assess the role of cognitive limitations in the context of work, a valid and easy-to-use self-report measure offers a practical advantage over timely and detailed neuro-psychological assessment. Self-report measures of cognitive function have been related to work output in cancer patients [10].

Several self-report instruments have been developed to measure cognitive symptoms in cancer patients [14, 15], including the Cognitive Symptom Checklist-100 (CSC-100). Based on the CSC-100, a 59-item work-specific cognitive symptoms questionnaire (CSC-W59) was developed [16]. To reduce the measurement burden, a 21-item version (CSC-W21) was developed and validated in working breast cancer survivors [17].

When using an established questionnaire in a different cultural context, it has to be translated, adapted and validated [18, 19]. Recently, the CSC-W21 has been translated and adapted for use in China [20]. The objective of this study is to cross-culturally translate and adapt the CSC-W21 into Dutch and to assess the reliability (i.e. internal consistency) and validity (i.e. structural and construct) of the CSC-W Dutch version (CSC-W DV). The COnsensus-based Standards for the selection of health status Measurement INstruments (COSMIN) taxonomy was used for the study design [21–23].

## Methods

### Cross-cultural translation and adaptation

A cross-cultural translation and adaptation of the CSC-W21 [18] was performed according to a standard systematic procedure, including forward translation, synthesis, back-translation, consolidation by an expert committee and pre-test [18, 19], ultimately leading to the CSC-W DV. During the translation and adaptation process, three original items (2 ‘difficulty remembering my train of thoughts as I am speaking’, 5 ‘difficulty remembering a word I wish to say’ and 6 ‘difficulty remembering the name of a familiar object or person’, Table 2) were discussed with the professional translator and the original author (MF) regarding the meaning of ‘remembering’. Two original items (9 ‘difficulty understanding a system’ and 12 ‘difficulty understanding systems and models’, Table 2) were difficult to distinguish due to conceptual overlap, and after consultation with MF, these items were combined.

A pre-test was performed with 13 cancer patients (age *M* = 51.2 years, *SD* = 6.0; 9 women) who had returned to work for at least 12 h a week after sickness absence. The CSC-W21 was developed to measure work-specific cognitive symptoms in cancer patients [18]. Working cancer patients were selected to explore the understandability, applicability and completeness of the items in working cancer patients. Participants had no difficulties in understanding the items and no concerns regarding applicability and completeness.

### Final questionnaire

The final CSC-W DV contained a set of 20 questions specific to a person’s work tasks and measured work-specific cognitive symptoms. To obtain a wider distribution in scores and to have a more responsive measure [24], the response options were changed from a dichotomous scale (i.e. yes or no) to an ordinal five-point scale, ranging from 0 = never to 4 = always. The response option ‘Does not apply to my job’ was added to enable cancer patients to answer, even though a particular symptom was not relevant to their job. Total scores were obtained by summing the scores on each item, divided by the number of items and then multiplied by 25 to obtain scores between 0 and 100, with higher scores indicating more cognitive symptoms (i.e. more limitations). The scores on ‘Does not apply to my job’ were transformed to missing values. If answers on 20 % or more of the items were missing, the total score was set to missing.

### Measurement properties of the CSC-W DV

#### Subjects and setting

The final version of the CSC-W DV was administered at baseline in the Work Life after Cancer (WOLICA) study. WOLICA is an ongoing longitudinal cohort study in the Netherlands, investigating cancer patients’ work functioning. Cancer patients aged 18–65 years, who had returned to paid work (during or following treatment for cancer) in the past 3 months for at least 12 h per week, and who had a history of paid work for at least 1 year prior to diagnosis, were eligible for WOLICA if able to complete a questionnaire in Dutch. Patients with recurrent cancer and patients treated with hospice care were excluded, because the study might be too burdensome for these cancer patients given the prognosis and the timeline of the study. Moreover, cancer patients in hospice care are not likely to be actively employed.

Occupational physicians (OPs; working at occupational health services) informed eligible cancer patients about WOLICA and forwarded the name and address of interested patients to the research team. The research team decided about the in- or exclusion of the participant. The cancer patients received an information letter, an informed consent form and the baseline questionnaire. Patients received no incentive for participation. All procedures were reviewed and approved by the Medical Ethical Committee of the University Medical Center Groningen (M12.125242).

#### Data collection

Participants provided information on age, gender, education (low = primary, junior secondary vocational and junior general secondary education; medium = senior secondary vocational education and senior general secondary education; high = higher professional education, college and university), cancer diagnosis and cancer treatment.

Work functioning was measured with the 27-item Work Role Functioning Questionnaire (WRFQ 2.0, *α* = 0.96) [25], designed to measure perceived difficulties in meeting work demands among workers given their physical health or emotional problems [25, 26]. The recall period is 4 weeks, and the response options range on a five-point scale from 0 = difficult all the time to 4 = difficult none of the time, with an additional response option ‘Does not apply to my job’. A total score was calculated by summing the scores on each item, divided by the number of items and then multiplied by 25 to obtain scores between 0 and 100, with higher scores indicating better work functioning. The scores on ‘Does not apply to my job’ were transformed to missing values. If more than 20 % of the items were left unanswered, scores were set as missing. Work functioning was classified as ‘high’ (>90), ‘medium’ (75–89) and ‘low’ (<75) [27].

Fatigue was measured with the 8-item subscale of the Checklist Individual Strength (CIS-8) [28, 29] designed to measure general severity of fatigue (*α* = 0.88). The recall period was 2 weeks with a seven-point response option scale from 1 = yes that is true to 7 = no that is not true. A total score was calculated by summing the items, with higher scores indicating more severe fatigue [28]. The total score was divided in tertiles.

Depression was measured with the 9-item Patient Health Questionnaire-9 (PHQ-9) [30, 31], designed to measure depression for non-psychiatric settings, matching the Diagnostic and Statistical Manual of Mental Disorders (DSM-IV) criteria of major depression. The recall period was 2 weeks, and the response options ranged from 0 = not at all to 3 = nearly every day. A total score was calculated by summing the items, and scores were dichotomized (1/0) as ‘low’ (<10) versus ‘high’ (10 or above, indicative of clinical depression) [30, 31].

General health was measured with the single SF-36 item ‘In general, how would you rate your health?’ [32]. The response options range on a five-point scale from 0 = excellent to 4 = poor. Scores were dichotomized (1/0) as ‘high’ (excellent/very good/good) versus ‘low’ (fair/poor).

### Statistical analysis

#### Structural validity

Exploratory factor analyses (EFA) were performed to examine the CSC-W DV subscale structure using principal component analysis (PCA) with oblique rotation (direct oblimin). A combination of the scree plot, eigenvalues, factor loadings and interpretation of the factors was used to decide on the number of factors. Items were explored for factor loading on its own factor (good if >0.5) and other factors (good if <0.3). Also, item inter-correlations were explored (ideal between >0.2 and <0.8) [24]. An item was excluded if it did not meet at least two of the above criteria and was not considered conceptually important.

#### Measurement characteristics

CSC-W DV mean scores, standard deviations (*SD*), range and percent at floor/ceiling were presented for the total score and subscales. If more than 15 % of the participants reported the lowest or highest scores, floor and ceiling effects were considered [33].

#### Reliability

CSC-W DV internal consistency was measured with Cronbach’s alpha, calculated for the total score and for each subscale (ideal >0.70) [24].

#### Construct validity

To determine the expected associations with related constructs, four hypotheses were assessed as follows:

1. Cancer patients with low work functioning report higher levels of cognitive symptoms than cancer patients with high work functioning.
2. Cancer patients with a high fatigue level report higher levels of cognitive symptoms than cancer patients with a low fatigue level.
3. Cancer patients with a high level of depressive symptoms report higher levels of cognitive symptoms than cancer patients with a low level of depressive symptoms.
4. Cancer patients with low self-rated health report higher levels of cognitive symptoms than cancer patients with high self-rated health.

Between-group differences were tested with *t* tests (two groups) or ANOVA (multiple groups). All analyses were performed with SPSS software (version 22).

## Results

### Sample characteristics

A total of 364 cancer patients participated in WOLICA; data of 341 (94 %) patients (61 % women) with a mean age of 50.6 (*SD* = 8.6) years who completed the CSC-W DV were included in the analyses. Most patients were diagnosed with breast cancer (45 %) or colorectal cancer (12 %). At inclusion, patients were diagnosed with cancer for 10.7 (*SD* = 6.3) months and almost 70 % (*n* = 234) had completed treatment (Table 1).

**Table 1:** Sample characteristics (*n* = 341)

Socio-demographics	
Gender, *n* (%)	
Male	132 (39)
Female	205 (61)
Age in years, *M* (*SD*)	50.6 (8.6)
Education, *n* (%)	
Low	80 (24)
Medium	120 (36)
High	134 (40)
Health characteristics	
Cancer type, *n* (%)	
Breast cancer	153 (45)
Colon cancer	40 (12)
Lymph node cancer	31 (9)
Prostate and testicular cancer	29 (9)
Other types of cancer	87 (25)
Months since diagnosis, *M* (*SD*)	10.7 (6.3)
Treatment completed, *n* (%)	
Yes	234 (69)
No	107 (31)
Type of treatment, *n* (%)	
Surgery	54 (17)
Chemotherapy	20 (6)
Surgery and chemotherapy	50 (15)
Surgery and radiation	41 (12)
Surgery, chemotherapy and radiation	66 (20)
Surgery, chemotherapy, radiation and hormonal therapy	47 (14)
Other combinations of treatment	53 (16)
WRFQ (range 0–100)	
*M* (*SD*)	77.7 (17.4)
High, *n* (%)	93 (29)
Medium, *n* (%)	111 (35)
Low, *n* (%)	114 (36)
Fatigue, *M* (*SD*) (range 8–56)	
Total (*n* = 290)	30.3 (11.4)
Low (*n* = 114)	17.6 (5.3)
Medium (*n* = 113)	30.6 (2.9)
High (*n* = 113)	43.0 (5.3)
Depressive symptoms (range 0–27)	
*M* (*SD*)	4.7 (3.8)
Low, *n* (%)	301 (88)
High, *n* (%)	40 (12)
Self-rated health, *n* (%)	
High	256 (76)
Low	79 (24)

*M* mean, *SD* standard deviation; *WRFQ* Work Role Functioning Questionnaire, *RTW* return to work

The median time back at work was 2 months. Patients were working on average 17.1 h (*SD* = 14.3) per week. Cancer patients reported that it was difficult to meet the demands of the job due to health problems 22 % of the time (WRFQ *M* = 77.7, *SD* = 17.4). Twenty-nine percent reported a WRFQ score of above 90, indicating little difficulty meeting work demands. The majority (76 %) reported good to excellent health. Cancer patients reported a mean fatigue score of 30.3 (*SD* = 11.4). Patients had a mean depression score of 4.7 (*SD* = 3.8), and 12 % (*n* = 40) reported relatively high depressive symptoms, indicative of clinical depression (Table 1).

### Structural validity

All 20 CSC-W DV items were included in an exploratory factor analysis (EFA) with 1 fixed factor. Reasonable total scale fit (factor loadings between 0.684 and 0.790) was observed. A three-factor solution did not replicate the original CSC-W21 three-factor structure [17]. A two-factor solution worked best (Table 2), with a ‘working memory’ subscale containing items from the original CSC-W21 ‘working memory’ subscale and an ‘executive function’ subscale containing items from the CSC-W21 ‘executive function’ and ‘task completion’ subscales. Because the item, ‘difficulty staying with a task until completion’, did not meet the inclusion criteria, it was removed. Consequently, the final CSC-W DV version has 19 items, with the subscales working memory (8 items) and executive function (11 items).

**Table 2:** Factor loadings Cognitive Symptom Checklist-Work Dutch version (*n* = 341)

CSC-W DV	CSC-W21	Items	Factor 1 working memory	Factor 2 executive function
1	1	I have difficulty remembering what I intended to write	*0.802*	0.052
2	2	I have difficulty remembering my train of thought as I am speaking	*0.841*	−0.045
3	3	I have difficulty remembering the content of telephone conversations	*0.841*	−0.001
4	4	I have difficulty remembering the content of conversations and/or meetings	*0.775*	0.102
5	5	I have difficulty remembering a word I wish to say	*0.816*	−0.037
6	6	I have difficulty remembering the name of a familiar object or person	*0.851*	−0.068
7	7	I have difficulty remembering information that is ‘on the tip of my tongue’	*0.796*	0.011
8	8	I have difficulty remembering things someone has asked me to do	*0.598*	0.256
9	9/12^a^	I have difficulty to understand that specific tasks along to a larger whole	−0.001	*0.774*
10	10	I have difficulty understanding how a task fits into a plan or system	−0.110	*0.890*
11	11	I have difficulty knowing where to look for information to solve a problem	−0.112	*0.917*
12	13	I have difficulty figuring out how a decision was reached	−0.198	*0.955*
13	14	I have difficulty using new information to re-evaluate what I know	0.095	*0.777*
14	15	I have difficulty considering all aspects of what I hear or see instead of focusing on only one part	0.195	*0.688*
15	16	I have difficulty understanding what a problem is when it occurs and clearly stating what the problem is	0.144	*0.651*
16	17	I have difficulty following the flow of events	0.356	*0.514*
17	18	I have difficulty understanding graphs or flowcharts	0.233	*0.587*
18	19	I have difficulty completing all steps of a task or activity	0.326	*0.543*
	20^b^	I have difficulty staying with a task until completion	0.458	0.350
19	21	I have difficulty putting steps in order such that the most important steps are done first	0.165	*0.637*

The italic numbers indicate items that grouped together in the final CSC-W DV. Extraction method: principal component analysis. Rotation method: Oblimin with Kaiser normalization

^a^Due to conceptual overlap, items 9 and 12 (original Cognitive Symptom Checklist–Work 21) were combined into item 9 (Cognitive Symptom Checklist-Work Dutch version, i.e. CSC-W DV)

^b^Not included in the CSC-W DV

### Measurement characteristics

The mean total scale score was 25.1 (*SD* = 15.8). Working memory had the highest scale score (*M* = 32.2, *SD* = 19.0). Floor and ceiling effects were not observed. Inter-item correlations were between 0.3 and 0.8. Executive function had the highest number of missing or ‘not applicable’ scores (Table 3).

**Table 3:** Measurement characteristics Cognitive Symptom Checklist-Work Dutch version (*n* = 341)

	Valid *n* (missing or ‘not applicable’)	Mean^a^ (*SD*)	Range (0–100)	*n* (%) at floor	*n* (%) at ceiling	Cronbach’s *α*
Working memory (8 items)	341 (0)	32.2 (19.0)	0–85.7	27 (8)	0 (0)	0.93
Executive function (11 items)	336 (5)	19.8 (15.8)	0–72.7	52 (15)	0 (0)	0.94
Total score (19 items)	341 (0)	25.1 (15.8)	0–76.5	20 (6)	0 (0)	0.95

^a^Each scale is scored from 0 to 100, with higher score indicating higher cognitive symptoms. Alphas calculated in SPSS (listwise deletion)

### Reliability

The Cronbach’s alpha for the total scale was 0.95 (Table 3).

### Construct validity

Cancer patients with low work functioning had higher CSC-W DV scores (indicating a greater number of cognitive problems at work) than those with high work functioning (Table 4). Cancer patients who reported a high fatigue level and/or depressive symptoms had higher CSC-W DV scores than those with a low level of fatigue and/or depressive symptoms. The CSC-W DV scores did not differ between cancer patients reporting low and high self-rated health.

**Table 4:** Comparing means (*n* = 341)

Variable	CSC-W DV, M (*SD*)	*P* value
Work functioning (WRFQ2.0) (*n* = 318)		
High (*n* = 93, 29 %)	14.1 (10.8)	<0.001^a^
Medium (*n* = 111, 35 %)	24.2 (13.1)	
Low (*n* = 114, 36 %)	35.4 (15.0)	
Fatigue (CIS) (*n* = 340)		
Low (*n* = 114, 34 %)	20.8 (13.9)	0.001^b^
Medium (*n* = 113, 33 %)	26.3 (15.8)	
High (*n* = 113, 33 %)	28.5 (16.6)	
Depressive symptoms (PHQ-9) (*n* = 341)		
Low (*n* = 301, 88 %)	23.2 (14.7)	<0.001
High (*n* = 40, 12 %)	39.6 (16.2)	
Perceived health (SF-36) (*n* = 335)		
High (*n* = 256, 76 %)	24.5 (14.8)	0.14
Low (*n* = 79, 24 %)	27.5 (18.4)	

*CSC-W DV*, Cognitive Symptom Checklist-Work, Dutch version, *WRFQ 2.0*, Work Role Functioning Questionnaire, *CIS* Checklist Individual Strengths, *PHQ-9* Patient Health Questionnaire-9, *SF-1* Short-Form 36

^a^Post Hoc analyses (Bonferroni) showed significant differences between all groups

^b^Post Hoc analyses (Bonferroni) showed no significant difference between the middle and high fatigue group

## Discussion

The original 21-item CSC-W21 was translated and adapted to the Dutch context by using a standardized systematic procedure for the translation and cross-cultural adaptation. This resulted in a final 19-item CSC-W Dutch version (CSC-W DV) with questions specific to a person’s work tasks, for measuring work-specific cognitive symptoms. The CSC-W DV contained two subscales (i.e. working memory and executive function) and showed good reliability without floor or ceiling effects. Three of four hypotheses to test the construct validity were confirmed, showing that the CSC-W DV was able to distinguish between groups with different levels of work functioning, fatigue and depression. No significant difference was found for self-rated health. The observation that almost all participants (94 %) completed the questionnaire suggests that the measure may have been seen as relevant and/or that the items were unambiguously formulated [22].

The main differences between the CSC-W DV and the existing CSC-W versions [17, 20] are the response options and the validation populations. The dichotomous response option was changed to an ordinal five-point scale. It was assumed that more response options would lead to a more refined self-evaluation of cognitive function at work [24]. Despite the differences in response set in the original and the cross-culturally adapted instrument, similar measurement properties (e.g. internal consistency and factor structure) were found. Although it is necessary to take this change in response set into account, the use of an expanded response set did not change the main measurement properties of the questionnaire and thereby, comparison of the data across languages is possible. In addition, it might be possible to dichotomize the answer categories for the comparison of data across languages. The three CSC-W versions were validated in populations with different types of cancer diagnosis, different case definitions and differences in cultures and languages. In the current study, cancer patients with all types of cancer were included during or following treatment, while the other versions were validated only in breast cancer patients following primary treatment [17, 20]. The CSC-W DV showed cognitive problems at work in a heterogeneous group of cancer patients at work, not only in breast cancer patients.

Despite the differences in versions and populations, similar measurement properties were found. The internal consistency and factor structure of the CSC-W DV were comparable despite different cultures and the fact that the items in the original subscales ‘executive function’ and ‘task completion’ in the CSC-W DV both loaded on ‘executive function’. Follow-up research could develop a shorter list, given the high Cronbach’s alpha’s and the fact that two items (i.e. original items 17 ‘difficulty following the flow of events’ and 19 ‘difficulty completing all steps of a task or activity’, Table 2) loaded high on both factors.

Patients with low work functioning reported higher CSC-W DV scores than cancer patients with high work functioning. This is in line with previous findings that higher scores on the CSC-W59 were correlated with higher scores on work output scales of the WLQ, indicating worse work outcomes in working breast cancer survivors [10]. We found that CSC-W DV scores were also related to fatigue and depression. According to Valentine et al., problems with cognitive functioning, fatigue and depression are very common in cancer patients [5]. Previous research in colorectal cancer patients also showed associations between self-reported cognitive function and fatigue, anxiety and depression [4]. Li et al., [6] reported fatigue in breast cancer patients as an important risk factor for perceived cognitive impairment.

Although cancer patients who reported low self-rated health reported more cognitive symptoms, the difference was not statistically significant when compared to patients with high self-rated health. This may indicate that cognitive functioning at work is not represented in self-rated health. An alternative explanation is that cancer patients with poorer health were excluded, because the inclusion criteria of the present study required that cancer patients had to work for >12 h a week. It is also possible that while a relationship exists among symptom burden measures of depression and anxiety and cognitive function, overall or general health is not necessary and sufficient for cognitive function at work. This needs to be further explored.

In this study, cancer patients were only included when they had returned to work. This means that the results of the current study are only generalizable to cancer patients at work and not to the true incidence of cognitive symptoms among cancer patients in general; this may be underestimated. Patients were diagnosed with various types of cancer and some cancer patients were on treatment, while others had completed treatment or received adjuvant therapy. The variation in treatment can be a study limitation because some adjuvant therapies can impact cognitive functioning [34]. However, this study was not designed to determine whether scores were related to type of treatment or a specific cancer. Future research is needed to examine the consistency of measurement properties of the CSC-W DV across cancer type and treatments. Another limitation is that we did not conduct concurrent neuro-psychological assessments, which may be a more objective standard to validate the CSC-W DV. Interestingly, Calvio et al. [10] observed that work-specific CSC scores were significantly related to work limitations, while neuropsychological test results were not. Furthermore, de Vet et al., [24] stated that in situations in which a gold standard is lacking, construct validation should be used to provide evidence of validity. Although self-report measures are known to have a low correlation with a neuro-psychological assessment of cognitive function in everyday life [3, 10, 35], it would be interesting to investigate the relationship between the CSC-W DV and neuro-psychological assessments approaches specifically developed to assess various types of cognitive functioning in problematic work tasks.

## Conclusion

This study indicates that the CSC-W DV can be used to measure cognitive symptoms in working patients with different cancer diagnoses. Knowledge about work-specific cognitive symptoms may improve guidance such as work accommodations to support cancer patients at work. To improve the quality of life and functioning at work, it is important to know which cognitive problems cancer patients experience. To make the CSC-W DV suitable for use on an individual level, it is recommended to develop cut-off points. Future comparative studies are now possible in three different countries with three different social systems and policies towards cancer and work. More information on the responsiveness of the CSC-W DV is needed when the CSC-W DV is used for monitoring cognitive function of working cancer patients and further longitudinal studies are needed to investigate the relationship between cancer diagnosis and cognitive functioning at work.
